# Impact of Heat on Birth Outcomes and Child Nutrition: Study Protocol using the CIDACS Birth Cohort

**DOI:** 10.12688/wellcomeopenres.23909.2

**Published:** 2025-08-28

**Authors:** Rita de Cássia Ribeiro-Silva, Maxine Pepper, Priscila Ribas de Farias Costa, Taisa Rodrigues Cortes, Lais Sacramento, Lais Helena Ribeiro, Lisianne Passos Luz, Otavio T. Ranzani, Liam Smeeth, Elizabeth B. Brickley, Aline dos Santos Rocha, Julia M. Pescarini, Ila Falcão, Poliana Rebouças, Danielson Delgado, Ismael Silveira, Enny S Paixão, Mauricio Barreto

**Affiliations:** 1 Universidade Federal da Bahia Escola de Nutricao, Salvador, State of Bahia, Brazil; 2 Instituto Goncalo Moniz, Salvador, State of Bahia, Brazil; 3 London School of Hygiene and Tropical Medicine Faculty of Epidemiology and Population Health, London, England, UK; 4 Instituto de Salud Global Barcelona, Barcelona, Catalonia, Spain; 5 Universidade Federal da Bahia Instituto de Saude Coletiva, Salvador, State of Bahia, Brazil

**Keywords:** Ambient temperature, Heat, Heatwaves, Birth outcomes, Child nutrition

## Abstract

**Background:**

Pregnant individuals and children are particularly vulnerable to the adverse health consequences of exposure to heat. Leveraging the robust ecosystem of Brazilian linked administrative data, we aim to advance our understanding of how high temperatures and heatwaves influence birth outcomes and child nutrition.

**Objectives:**

Investigate the association between high ambient temperatures/ heatwaves and 1) birth outcomes (low birth weight (LBW), small for gestational age (SGA), large for gestational age (LGA), and preterm birth), 2) child nutrition (growth), and 3) breastfeeding practices and complementary feeding in children.

**Methods:**

We will triangulate results from complementary analytical approaches, including time-stratified case-crossover designs and time-to-event techniques. We will explore the influence of high temperatures and heatwaves at different periods (or lags) preceding the event and apply distributed lag non-linear models to account for delayed and non-linear effects. We will conduct subgroup analyses to identify the population groups most at risk.

**Results and Conclusions:**

Our study will generate new insights into the relationship between heat, birth outcomes, and child nutrition in Brazil. By providing evidence from a middle-income country with diverse ecosystems and climate zones in a context of social inequalities, this research will contribute to advancing the current knowledge base. Additionally, by identifying critical windows of vulnerability and at-risk groups, our findings can potentially inform targeted and equitable climate adaptation policies.

## Plain summary

Pregnant people and young children are particularly vulnerable to the health impacts of high temperatures. This study uses detailed data routinely collected in Brazil to investigate how high temperatures are associated with outcomes such as babies being born too small or too soon, and how they affect young children's growth and nutrition. Additionally, we will examine how heat influences breastfeeding practices and food intake. To analyse this, we will use advanced statistical methods to understand both immediate and delayed effects of heat exposure and identify the groups most at risk. By studying Brazil, a large middle-income country with diverse climates, and a high level of social inequalities, we aim to provide new insights into the health impacts of high temperatures. Our findings will inform the development of policies that address these risks in an equitable and effective manner, generating evidence to guide policies for the protection of the populations most vulnerable to heat.

## Introduction

The rapid increase in greenhouse gas emissions driven by human activities has led to substantial rise in the average global temperature, with the past decade being the warmest in history ^1^. Prolonged exposure to extreme temperatures presents both direct and indirect health risks, particularly to vulnerable populations such as pregnant women, newborns, and children ^2^. For example, exposure to elevated temperatures during pregnancy can result in physiological changes such as reduced placental blood flow due to vasodilation and inflammatory responses that may trigger early labour ^3^. These changes are linked to an increased risk of adverse birth outcomes, including preterm birth, which remains the leading global cause of neonatal and under-five child mortality ^4^.

In children, exposure to high ambient temperatures has been associated with a range of acute health issues, including heat stress-induced appetite loss and dehydration ^5^. These conditions can impair nutrient retention and absorption, resulting in acute malnutrition characterized by weight loss ^6^. Prolonged nutritional deficiencies may lead to chronic malnutrition and stunted growth ^7^. Elevated temperatures may also indirectly exacerbate underlying determinants of health by reducing agricultural yields and limiting water availability, contributing to food insecurity and compromised nutritional intake ^5^.

High temperatures may also impact dietary practices, including breastfeeding ^8^. In the context of water insecurity, heat increases the risk of dehydration, raising concerns about its potential effects on lactation ^9^. However, the effects of dehydration on lactation in hot climates remain largely unknown. Evidence suggests that exclusively breastfed infants generally maintain adequate hydration in warm conditions, implying that breast milk production may not be significantly affected ^10, 11^. Nonetheless, experimental animal study indicates that chronic exposure to high temperatures (e.g., 41°C) could impair the milk-producing capacity and reduce the number of mammary epithelial cells ^12^. Although no definitive evidence confirms that maternal milk supply is adversely affected, elevated temperatures may influence mothers' perceptions of milk sufficiency. These perceptions can, in turn, affect the timing of introduction of water and complementary foods, which may influence child growth and development.

The effects of extreme high temperatures have undermined progress in improving child health, with the burden disproportionately affecting the most vulnerable populations. These impacts are often exacerbated by existing inequalities and compounded by interactions with other crises. While populations in low- and middle-income countries (LMICs) are particularly vulnerable to the health consequences of rising temperatures ^13^, most research on heat and birth outcomes is conducted in high-income countries, focusing on populations in temperate climates with greater access to mitigation strategies and adaptive capacities ^14^.

There is an urgent need to build climate resilience, including enhancing preparedness for climate-related emergencies, and to develop effective adaptation strategies for those most at risk. This includes prioritizing individuals living in poverty, particularly those in extreme poverty and socially or ethnically marginalized groups. To achieve this, it is crucial to generate more evidence-based research from LMICs, with analyses stratified by at-risk groups, to inform targeted and equitable climate adaptation policies.

The Center for Data Integration and Knowledge in Health ( *Centro de Integração de Dados e Conhecimentos para Saúde,* CIDACS) has significant potential to advance research on the effects of temperature on birth outcomes and child health by leveraging its robust ecosystem of Brazilian linked administrative data. This includes data from the CIDACS Birth Cohort and the recently launched CIDACS-Clima Climate and Health Data Platform. Using these nationally representative, longitudinal data resources from a large LMIC country is expected to provide critical insights and evidence to support the implementation of adaptation measures that address the adverse health consequences of rising temperatures.

## Aims and objectives

- To estimate the association of high ambient temperature and heatwaves on birth outcomes, including low birth weight, small for gestational age (SGA), large for gestational age (LGA), and preterm birth, in the general population and within specific subgroups of interest;
- To estimate the association of high ambient temperature and heatwaves on child nutrition, both in the general population and within specific subgroups of interest;
- To evaluate the influence of high ambient temperature and heatwaves on breastfeeding practices and complementary feeding in children.

## Conceptual framework

Our conceptual framework recognizes the complex interplay between multiple mechanisms which may underlie the relationship between high ambient temperatures and heat waves, adverse birth outcomes, and growth faltering in infants and young children. In this protocol, we do not propose novel pathways; this framework builds on existing literature.

Evidence indicates that prenatal exposure to elevated temperatures can have trimester-specific effects on placenta and embryogenesis ^15^. In the first trimester, such exposure has been associated with epigenetic modifications as well as physiological, cellular, and metabolic changes. During the second and third trimesters, heat exposure may induce oxidative stress, stimulate the release of pro-inflammatory cytokines, and increase the expression of heat shock proteins – physiological responses which have been implicated in the initiation of early labour and preterm birth ^3^.

The influence of elevated temperatures on placental capacity and blood flow remains an area of ongoing investigation, with various proposed mechanisms suggesting divergent – sometimes opposing – impacts on adverse birth outcomes such as SGA or LGA. Some studies report reduced placental blood flow and impaired placental function, potentially contributing to the risk of SGA births. In contrast, other studies suggest that exposure to high temperatures in late pregnancy may influence maternal vascular function and nutrient delivery to the fetus ^16^, or enhance placental performance and trigger beneficial metabolic adaptations, thereby ultimately supporting fetal growth and resulting in higher birth weight ^17^.

Additionally, during periods of extreme heat, pregnant individuals are often advised to increase fluid intake to prevent dehydration. However, this may inadvertently lead to greater consumption of both sugary and non-sugary beverages, raising concerns about potential adverse effects on maternal and fetal health, such as increased risk of gestational diabetes, excessive maternal weight gain, fetal macrosomia, and LGA births ^18^.

Moreover, physical activity tends to decline in the later stages of pregnancy ^19, 20^, a reduction that may be further exacerbated by high temperatures ^17, 18^. Elevated ambient temperatures have also been associated with increased maternal leptin levels ^19^, which may contribute to higher birth weight ^20^.

Given the mixed evidence and the ongoing nature of research in this area, our investigation will consider both SGA and LGA outcomes to capture the full spectrum of potential fetal growth responses to prenatal heat exposure. SGA, LGA, and preterm birth are all critical adverse birth outcomes which have lasting consequences for infant growth ^21^.

For infants and young children, exposure to extremely high temperatures and heat waves are linked to an increased risk of wasting and underweight through various pathways. Hypothesized mechanisms include reduced food intake (including breastmilk due to altered feeding practices), alterations to the gut microbiome, which may be associated with increased risk of diarrheal episodes and diminished nutrient absorption. Food insecurity linked to heat and drought conditions, which may negatively affect crop yields, further exacerbates these risks. The mechanisms contributing to stunting are more complex than those for wasting and often involve indirect pathways such as food insecurity, decreased water availability, and weakened health systems, which can hinder vaccination programs and essential health services, all of which contribute to child malnutrition ^6^.

**Figure 1.**
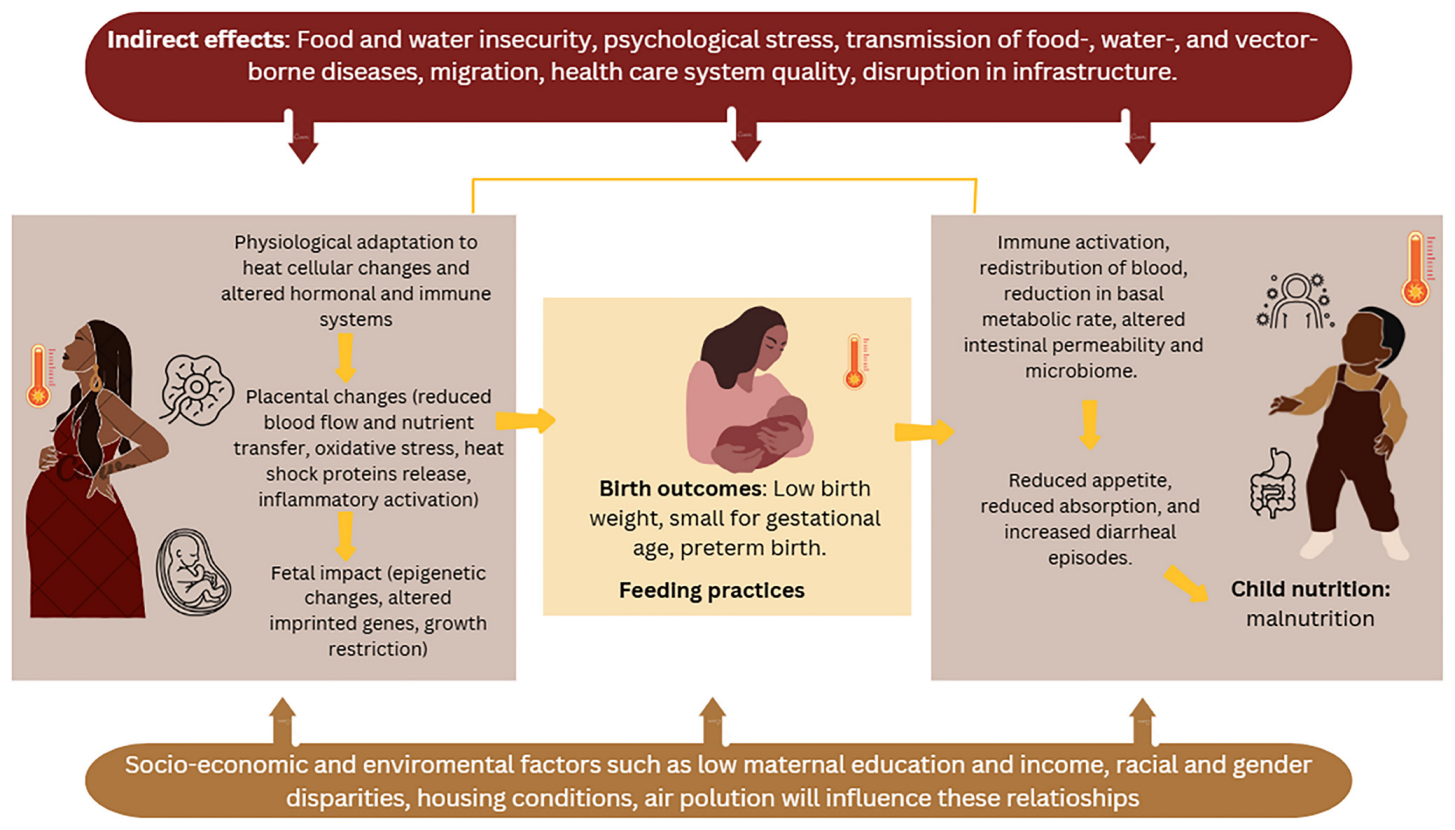
Theoretical framework guiding the investigation of high ambient temperature and heatwaves of birth outcomes, feeding practices and child nutrition.

These complex relationships have been insufficiently studied in LMICs. The health impacts of climate change are multifaceted, varying across geographic regions and socioeconomic contexts, and are likely to be exacerbated by existing social inequalities. While some outcomes have short-term windows of susceptibility, others reflect the delayed effects of exposure to (prolonged) heat. However, further research is needed to better define these critical windows of vulnerability ( Figure 1).

## Methods

### Study population and data sources

This protocol was developed following the Reporting of Studies Conducted Using Observational Routinely Collected Health Data (RECORD) guidelines ^22^. We will use the CIDACS Birth Cohort ^23^. This cohort (N=28,631,394 liveborn children 2001–2018) was established to investigate the impact of prenatal and early life events on health-related outcomes for infants, children, adolescents, and pregnant individuals in the context of social inequalities. A detailed description of the cohort and the list of datasets linked to this data resource can be found in Paixão *et al.* (2021) ^23^. It was built through the linkage between:

I) Socioeconomic and demographic data from the National Unified Register for Social Programmes (CadÚnico, Cadastro Único), CadÚnico identifies low-income families applying for social assistance in Brazil. The baseline dataset includes 131,697,800 individuals, approximately 62% of the Brazilian population, who entered the cohort during different periods (2001–2018). The CadUnico database originates from an extensive questionnaire completed at the time of application to federal social programs. It contains detailed demographic, economic, and social data at the household and individual levels ^24^.
II) Live birth records, including information from the Live Birth Information System (SINASC, Sistema de Informação sobre Nascidos Vivos); SINASC is the primary national system recording the data available in the "declaration of live birth", legally required for every live birth in Brazil. The document is filled by the health worker who assisted the childbirth immediately after birth. In cases where the delivery was not assisted or performed by a registered professional, the declaration is filled in by the Civil Registry Office. The declaration form includes data on maternal characteristics, pregnancy and delivery ^25^.

The birth cohort was linked to data from the Food and Nutrition Surveillance System (SISVAN, Sistema de Segurança Alimentar e Nutricional) ^23^:

III) SISVAN is a national surveillance system designed to collect data on individual anthropometric indicators and food intake, based on information recorded by Primary Health Care professionals during routine services in the public healthcare system (SUS, Sistema Único de Saúde). These professionals are responsible for delivering healthcare and conducting nutritional surveillance across all stages of life, including monitoring the nutritional status of children enrolled in the Bolsa Família conditional cash transfer program ^26, 27^. Between 2008 and 2017, SISVAN coverage among the SUS-using population increased from 17.7% to 45.4%. Coverage of the total resident population rose from 14.3% to 34.6% over the same period ^26^. The main objective of SISVAN is to support the evaluation and development of public health nutrition policies. The dataset available at CIDACS includes 307,245,508 records from 59,724,164 individuals, covering the period from 2008 to 2021 ^26^

The climatic variables at municipality level were extracted from two publicly available gridded datasets:

IV) The ERA5-Land reanalysis dataset from the European Centre for Medium-Range Weather Forecasts' (ECMWFs'); ERA5-Land is a global atmospheric reanalysis for land areas produced by running only the terrestrial component of the ERA5 climate reanalysis model from ECMWF. A spatial subset of the global reanalysis was obtained for Brazil’s continental area at a 0.1° x 0.1° (~9 km ^2^) spatial resolution and hourly frequency ^28^. ERA5-Land combines observations and model data to provide consistent spatial and temporal coverage at a global scale. Variables used included hourly 2m air temperature and 2m dew point temperature, from which it was possible to calculate the daily minimum, mean and maximum air temperature and the relative humidity derived by combining the prime variables.
  V) The Brazilian Daily Weather Gridded Data (BR-DWGD): The BR-DWGD is a gridded meteorological dataset produced by means of interpolation of observed data from 11,473 rain gauges and 1,252 weather stations across Brazil, provided by the Agência Nacional de Aguas (ANA) and Instituto Nacional de Meteorologia (INMET). These interpolated data were obtained from a publicly available archive. BR-DWGD provides several variables at a daily time resolution and high spatial resolution of 0.1° x 0.1° by including data on topographic relief in the interpolation process. Variables used included minimum and maximum temperatures and relative humidity.

We will use two climatic datasets due to their complementary strengths. The choice of primary dataset for each analysis will depend on the specific study objectives, data needs, and target audience. The BR-DWGD is widely recognised within Brazil and supports engagement with national stakeholders, while ERA5-Land facilitates comparability with international studies ^29, 30^. Ongoing validation work will also inform this decision, and both datasets may be used in sensitivity analyses where appropriate.

### Data linkage

Two approaches have been employed to perform record linkage between the data sources: **(a)** deterministic linkage and **(b)** non-deterministic linkage based on a similarity index.

The deterministic approach uses a unique identifier common in the two linked datasets. For example, climatic data from the CIDACS Climate and Health Platform was incorporated into the cohort database through deterministic linkage, using the municipality code of the mother's residence as a common identifier between datasets. To link the CIDACS Birth Cohort baseline with the SISVAN dataset, deterministic linkage was performed using a unique identifier available in both datasets – the Social Information Number (NIS, Número de Identificação Social). For records with missing NIS data, a non-deterministic linkage approach, similar to the method used to create the CIDACS Birth Cohort, was applied. The non-deterministic linkage process used to create the CIDACS Birth Cohort is described in detail by Almeida *et al.* ^31^. Matching criteria included variables such as maternal name, date of birth or age, and place of residence. This linkage was conducted using CIDACS-Record Linkage, an innovative tool developed at the Center for Data and Knowledge Integration for Health (CIDACS-RL) ^32^. CIDACS-RL utilizes a combination of indexing and search algorithms to identify records in the CIDACS Birth Cohort that closely match each record in the other datasets. The tool conducts pairwise comparisons of candidate linking records, ranks them based on similarity scores, and retains the highest-scoring pair as the potential link. The accuracy of each linkage will be assessed and described through manual verification of a random sample of records, evaluating sensitivity and specificity indexes via receiver operating characteristic curves. Before analysis, the final linked dataset will undergo a comprehensive data structuring process.

### Exposures

Information on exposure to high ambient temperatures and heatwaves will be assessed at the municipality level. This will be derived by calculating the area-weighted average of values from the geographical cells that intersect each municipality, with weights based on the proportion of the municipality area covered by each cell. The exposures will be defined and operationalized as follows:

1. Ambient air temperature: for each municipality, we will extract daily mean, minimum, and maximum ambient air temperatures (°C). We will compare exposure to high temperatures (95 ^th^ percentile) to the median temperature value (50 ^th^ percentile of the municipality-specific baseline temperature distribution). Moreover, we will also investigate exposure to very high temperatures (99 ^th^ percentiles; with the 75 ^th^ or 50 ^th^ percentile as reference values) ^33^.
2. Heatwaves: adopting the definition used by the *Lancet Countdown*, we will define heatwaves as a period of 2 or more consecutive days on which both minimum and maximum daily temperatures were above the 95 ^th^ percentile of the municipality-specific baseline temperature distribution ^34^. Recognizing the absence of a universally accepted definition ^35^, we will also define heatwave events by combining other temperature intensity criteria (i.e., 90 ^th^ and 99 ^th^ percentile) and different durations (i.e., at least 3 or 4 consecutive days) ^36^.

### Outcomes

The main outcome variables considered in this study protocol are:

**Child and mother variables**

Gestational age at birth [GA]: Categorized: [(<22, 22 to 27, 28 to 31, 32 to 36, 37 to 41, and 42 or more weeks ^37^; (discrete variable in complete weeks)].

Birth weight [BW]: Categorized: (Low birth weight (<2500g) and Macrosomia (>4000g) ^37^; (Very low birth weight (<1500g) and extremely low birth weight (<1000g) ^37^; (continuous variable, in grams)].

Birth size, estimated using gestational age at birth and birth weight: Categorized: [Large (> percentile 90) and Small (< percentile 10) for gestational age], according to Intergrowth-21st sex and gestational age-specific charts ^38^;

Body Mass Index (BMI) up to 5 years old estimated using weight and height: Categorized: [(BMI-for-age z-scores for sex according to the WHO charts will be used ^39^; (Underweight (BMI-for-age<-2 z-score); Normal (BMI-for-age≥-2 and ≤ 2 z-score); Overweight (BMI-for-age>2 and ≤ 3 z-score); Obesity (BMI-for-age>3 z-score)]; (continuous variable, in kg/m2))].

Nutritional status - Height for age z-scores [HAZ], Weight for-age z-score [WAZ] and Weight for height z-scores [WHZ]. Categorized as proposed by the Brazilian Ministry of Health ^40^: [(Stunting (HAZ <-2 z-score); (Normal (HAZ ≥-2); (Underweight (WAZ for age <-2 z-score); (Normal (WAZ ≥-2 and ≤3 z-score); (Obesity (WAZ for-age >3 z-score). (Wasting (WHZ <-2 z-score); (Normal (WHZ>-2); (continuous variable, in z scores)].

SISVAN Food Intake Markers: For children < 6 months old: consumption of breast milk; porridge; water/ tea; cow milk; infant formula; fruit juice; fruits; other food/ beverages. For children 6 – 23 months old: consumption of breast milk; whole fruit in pieces or mashed (and the daily frequency); food (and the daily frequency and how the food was offered); milk other than breast milk; porridge; yogurt; vegetables; or fruit or dark green leaves (spinach, kale); leaf greenery (lettuce, cabbage); meat (beef, chicken, pork, fish) or eggs; liver; beans; rice, root vegetable or pasta; hamburger and/or sausages; sugar-sweetened beverages; instant noodles, packaged snacks, or crackers; biscuits/cookies or candies. For children > 24 months old: beans; fresh fruits (excluding fruit juice); vegetables and/or greens (excluding potatoes, cassava, yams, taro, and sweet potatoes); sweetened beverages (soft drinks, boxed juice, fruit juice with added sugar); instant noodles, packaged snacks or savory biscuits; and filled biscuits, sweets.

The response options are: "yes," "no," and "do not know” if consumed in the previous day.

Food consumption records from SISVAN will be used to calculate the following child feeding practice indicators: Duration of exclusive breastfeeding defined as the length of time, from birth until the last recorded visit, during which children up to 5 months and 29 days were reported to be receiving only breast milk; duration of continued breastfeeding defined as the length of time, from birth until the last recorded visit, during which children aged 6 to 23 months and 29 days were reported to be receiving breast milk.; introduction of solid, semi-solid or soft foods (ISSSF); minimum meal frequency; minimum dietary diversity (MDD); minimum acceptable diet (MAD); consumption of meat and/or eggs; consumption of sweetened beverages; consumption of ultra-processed foods (UPF); and zero consumption of fruit and vegetables ^41, 42^.

 These variables will be outcomes for some objectives proposed but can also be confounders or effect modifiers for other objectives. Whenever possible, we plan to explore outcomes as both continuous and categorical variables, according to the proposed aim.

### Analytical approach

For most of the outcomes under study, the existing evidence base is limited, making it difficult to predefine key methodological elements such as the shape of the exposure-response relationship and appropriate lag periods. Additionally, some analyses will focus on short-term exposure (e.g., within the week preceding the outcome), while others will explore longer lag periods—up to one year—to identify potential windows of susceptibility. Given that heat exposure may have both acute and chronic effects on child nutrition, there is currently no consensus on the optimal lag period for assessing its impact on undernutrition. Some studies have adopted a one-year lag, arguing that a longer time window is more appropriate for capturing both immediate and cumulative effects of temperature, while also accounting for seasonal variation. In line with this rationale, we plan to use a 52-week lag period to assess potential medium- to long-term associations between ambient temperature exposure and children's nutritional status.

Therefore, this protocol outlines a general analytical approach. Detailed analysis plans, including specifications for sensitivity analyses, will be developed for each study and made available in an online repository.

For each analysis, we plan to construct directed acyclic graphs (DAGs) with the program dagitty ( http://www.dagitty.net/) and to include confounders defined *a priori*. The identification of potential confounders and effect modifiers will be guided by existing literature and the substantive expertise of the research team and collaborators. Confounders will include both environmental factors (e.g., humidity) and individual-level sociodemographic variables (e.g., maternal education). Although the exposure will be measured at the ecological level, it will be considered as a proxy for individual-level exposure. Consequently, we will adjust for individual-level covariates in the analysis, when appropriate based on the study design. Adjustment for seasonality and long-term time trends will be accounted for by design (e.g., by conducting case-crossover analyses) or by including covariates in the model with time terms, allowing for non-linearity. DAGs will also be used to identify potential residual confounding, which will inform sensitivity analyses to ensure robust findings.

We will triangulate findings from complementary methodological approaches to address our research objectives and investigate the influence of high temperatures and heatwaves across various time periods (or lags) leading up to the event. To capture non-linear and delayed effects of high ambient temperature/heatwaves, we will integrate distributed lag nonlinear models (DLNMs) into our analyses ^43^. Applying DLNMs involves defining a cross-basis which describes the exposure-response relationship along two dimensions: the predictor space and the lag dimension. The specification of the cross-basis will be informed by existing evidence about the temperature-outcome relationship, where possible. We will also use the Akaike Information Criterion (AIC) to guide model selection, with lower AIC values indicating the preferred model fit. To test the robustness of our findings, we will conduct sensitivity analyses with different cross-basis specifications, when appropriate.

DLNMs will be integrated into a suite of complementary methodological approaches based on case-crossover and time-to-event analyses. Additionally, other models and study designs may be incorporated as needed.

1. Case-crossover studies: this design will be used to study the effect of short-term exposure on acute events (e.g., preterm birth). In case-crossover studies, only individuals with the outcome are included and the exposure level on the day of the health event (“case day”) is compared with exposure levels on proximate days when the health event did not occur (“control days”). We will adopt the time-stratified approach and select control days within the same year, same month, and on the same day of the week as the case day ^44^. The case-crossover design controls confounding factors that are stable (or vary slowly) over time (e.g., individual characteristics such as race/ethnicity or education) by design. However, we will adjust for time-varying confounders such as relative humidity. As a primary statistical model, we plan to use conditional Poisson regression, which will allow us to adjust for overdispersion using quasi-likelihood functions ^45^.
2. Time-to-event analyses: This design leverages the longitudinal structure of the data and is particularly well-suited for handling censored observations. It is ideal for scenarios where accounting for the timing of an event is critical, such as gestational age at birth (e.g., preterm delivery) or the time elapsed since childbirth (e.g., feeding practices). We will use gestational age or time since childbirth as the timescales ^46^. Aligning exposure data with the at-risk window breaks the dependence of exposure assignment and gestational age/time since childbirth, thereby preventing immortal time bias ^46^. This is important where the likelihood of an outcome increases with gestational age or age of the child and where time trends in ambient temperature exposure (i.e., seasonality and long-term trends) cause average exposure over longer windows to systematically differ from exposure over shorter windows ^47^. Models can be fitted with time-dependent exposures with short (e.g., lag: 0–2 days) or longer lag structures (e.g., lag: 0–4 weeks) ^45^. Using this approach, we can investigate critical windows of exposure across the entire pregnancy and/or the child’s lifespan using a modified version of an approach suggested by Leung *et al.* ^46^. Our approach will consist of the following steps:
  a. Define the start of follow-up as conception or birth and split the data into different risk sets for each week of follow-up.
  b. For each risk set, fit a DLMN with weekly (or monthly) lags ranging from the start of follow-up to the risk set. This allows the length of the exposure window to increase over the course of follow-up.
  c. Extract the lag-specific risk estimates for each risk set ^43^.
  d. Combine the lag-specific risk estimates across risk sets using a meta-analysis. Lag-specific risk estimates need to be aligned at the start of follow-up to ensure that they correspond to the same window of exposure (e.g., gestational week 1).
3. Other models: We might also apply the DLNM approach to model the matrix of exposure history in other models, such as general linear models, generalized linear mixed models and generalized additive models, inside the labelled DLNM extended applications. For example, to estimate the effect of heat on breastfeeding duration, we will use linear regression models to estimate the effect size (β) and corresponding 95% confidence intervals (95% CI), using median temperature as the reference category. We will follow the applications in the literature, accounting for confounders and seasonality/long-term time trends.

Brazil has approximately 5,570 municipalities, which vary, for example, in terms of size and climate. To account for this heterogeneity, independent of the methodological approach, we will adopt a two-stage design, which is a common method for the analysis of multi-location data within environmental research ^48– 50^. In the first stage, we will estimate location-specific exposure-response associations (at the municipality level). In the second stage, we will pool the estimates to the regional level using meta-analytical methods. Alternatively, we might use alternative ways to aggregate such as Köppen climate classification to pool effect estimates ^51^. We will also explore other methods which have been suggested for multi-location studies (e.g., the space-time-stratified design for case-crossover studies and case times series) ^44, 52^.

We will conduct a series of subgroup analyses to characterize those women and children at greatest risk of the adverse health consequences of high temperatures. As the data allow, we will stratify by maternal characteristics (e.g., education level, race/ethnicity, gestational age at delivery), infant characteristics (e.g., sex, age and birth weight categories) and family characteristics (e.g., area of residence – rural/urban, beneficiary or not of social programs) and test for interaction effects with the exposure.

The proposed analyses will be conducted using R or Stata.

### Limitations

This study relies on routinely collected administrative data, which enables national coverage and a large sample size, but also presents several limitations. First, some outcomes are only recorded when individuals seek healthcare. For example, SISVAN data on food consumption and nutritional status are collected during visits to primary care facilities, introducing potential selection bias related to healthcare-seeking behaviour. Moreover, individuals with malnutrition or those at risk of adverse nutritional outcomes are more likely to have multiple evaluations recorded in this dataset and approximately 68% of children in SISVAN are Bolsa Família beneficiaries, due to a program conditionality requiring nutritional monitoring for children aged 0–7. This results in an overrepresentation of socioeconomically vulnerable groups, further limiting the generalisability of findings derived from SISVAN. More broadly, generalisability is also constrained by the composition of the CIDACS Birth Cohort, which includes only individuals registered in CadÚnico, representing the poorest half of the Brazilian population ^23^. Second, the use of administrative data restricts the availability of key covariates, such as maternal comorbidities, parental smoking, or household characteristics like access to air conditioning. Lastly, administrative data are subject to missingness, underreporting, and misclassification. For example, preterm births are estimated to be underreported by around 15% in SINASC, and gestational age may be misclassified due to reliance on the date of last menstrual period ^53^

Furthermore, there are also limitations related to the exposure data. Temperature was assessed at the municipality level, which may lead to measurement error due to the inability to capture within-municipality variation, such as urban heat island effects. However, prior studies on mortality have shown that using city-level average temperatures produces results similar to those obtained with more spatially resolved models ^54, 55^ suggesting that municipality-level exposure may still provide reasonable estimates . In future work, finer-scale exposure data – such as at the census tract level – may become available through the CIDACS Climate and Health Platform, offering opportunities for more spatially refined analyses, exploring spatial correlation within a Bayesian framework, such as the recently formalised spatial Bayesian DLNM approach.

## Ethics approval and consent to participate

The objectives described in this protocol are subprojects of the 100 Million Brazilian Cohort and CIDACS Birth Cohort, which was approved by the Research Ethics Committees of the Gonçalo Moniz Institute, Oswaldo Cruz Foundation (CAAE: 56003716.0.0000.0040) and [CAAE: 18022319.4.0000.5030]. As the study will exclusively use routinely collected and de-identified data, in accordance with Resolution no. 466/2012 established by the Brazil National Health Council’s Commission for Ethics in Research Brazil, the requirement for study participants to provide informed consent is waived. The objectives described in this protocol were approved by the Research Ethics Committee from Nutrition School/Federal University of Bahia (CAAE: 76672823.8.0000.5023; V2). 

## Consent for publication

Not applicable.

## Data Availability

The data that will support the findings derived from this study protocol are available from the Centre for Data and Knowledge Integration for Health (CIDACS), Oswaldo Cruz Foundation, but restrictions apply to the availability of these data, which will be used under license for the current study protocol, and so are not publicly available. Data are however available upon reasonable request (using this email: cidacs.comunicacao@fiocruz.br) and with permission of CIDACS. *Bolsa Familia is the conditional cash transfer program implemented in Brazil since 2004. ** In 2010, the system's coverage was estimated to be 95% in some Brazilian regions (58).
