# Supplementary material for: Impact of Heat on Birth Outcomes and Child Nutrition: Study Protocol using the CIDACS Birth Cohort
Source: Wellcome Open Res. 2025 Aug 28;10:169. Originally published 2025 Apr 1. [Version 2] doi: 10.12688/wellcomeopenres.23909.2 (PMC12461186; doi:10.12688/wellcomeopenres.23909.2)
Supplement: Peer review history

# Reviewer report: Reviewer response for version 2

**DOI:** 10.21956/wellcomeopenres.27286.r132426

**Published:** 2025-09-25

**Authors:** Sohail Lakhani

**Affiliations:** 1 The Aga Khan University, Karachi, Sindh, Pakistan; 2 Epidemiology & Biostatistics, Texas A&M University School of Public Health (Ringgold ID: 26515), College Station, Texas, USA

This study aims to analyse the relationship between ambient heat and, pregnancy outcomes and child nutrition by doing secondary analysis of CIDACS birth cohort. The robust methodology will allow for a comprehensive assessment of how heat exposure influences these outcomes. However, 

 1. While the study is bridging the gap of evidence in LMICs, it would be beneficial to reference the systematic reviews and studies conducted in LMICs in this area to strengthen the rationale and make it more meaningful and relevant. 

 2. Instead of solely relying on ambient temperature, using heat indices that account for temperature and humidity would be more meaningful as they reflect the perceived (feels like) temperature rather than just the ambient.

 Overall, incorporating these suggestions would enhance the impact and relevance of study and make its findings even more robust and applicable.

Is the study design appropriate for the research question?

Yes

Is the rationale for, and objectives of, the study clearly described?

Yes

Are sufficient details of the methods provided to allow replication by others?

Yes

Are the datasets clearly presented in a useable and accessible format?

Yes

Reviewer Expertise:

Heat and pregnancy outcomes

I confirm that I have read this submission and believe that I have an appropriate level of expertise to confirm that it is of an acceptable scientific standard, however I have significant reservations, as outlined above.

# Reviewer report: Reviewer response for version 2

**DOI:** 10.21956/wellcomeopenres.27286.r130878

**Published:** 2025-09-04

**Authors:** Ana Bonell

**Affiliations:** 1 London School of Hygiene and Tropical Medicine Faculty of Epidemiology and Population Health, London, UK

Thank you for addressing all the points raised.

Is the study design appropriate for the research question?

Yes

Is the rationale for, and objectives of, the study clearly described?

Yes

Are sufficient details of the methods provided to allow replication by others?

Partly

Are the datasets clearly presented in a useable and accessible format?

Not applicable

Reviewer Expertise:

Maternal health and climate change.

I confirm that I have read this submission and believe that I have an appropriate level of expertise to confirm that it is of an acceptable scientific standard.

# Reviewer report: Reviewer response for version 1

**DOI:** 10.21956/wellcomeopenres.26377.r123928

**Published:** 2025-06-23

**Authors:** John Molitor

**Affiliations:** 1 Oregon State University, Corvallis, Oregon, USA

This study protocol outlines a highly promising and timely investigation into the health impacts of heat exposure on birth outcomes, child nutrition, and feeding practices in Brazil. The research team leverages a remarkable array of linked administrative datasets, and the analytical plan demonstrates thoughtful consideration of time-varying exposures and lagged effects. The project is especially important in the context of low- and middle-income countries, where such large-scale evidence is scarce yet urgently needed.

 The proposed methodology is largely sound and appropriately ambitious, including use of distributed lag non-linear models (DLNMs), case-crossover designs, and time-to-event analyses. However, one critical limitation is the absence of spatial statistical modeling. The current approach aggregates exposures at the municipality level, but this overlooks two important concerns: spatial correlation in both exposures and outcomes and how these correlations affect associations.

 To address this, the authors should consider spatially varying coefficient (SVC) models, ideally within the R-INLA framework, which is well-suited for large-scale hierarchical models with spatial components. Incorporating spatially structured random effects (e.g., using BYM2) or allowing exposure effects to vary geographically would offer two major advantages: First, it would account for spatial dependence and unmeasured area-level confounding; second, it would allow the team to detect regional variation in the heat-health relationship—variation that is likely to be highly relevant for targeted adaptation policy. Given the availability of rich spatial data through CIDACS, this is a missed opportunity and should be considered as and addition to the analysis strategy.

 The authors state: "Two approaches have been employed to perform record linkage between the data sources: (a) deterministic linkage and (b) probabilistic linkage based on a similarity index." Only the deterministic linkage method appears to be described in the protocol; it would be helpful to clarify whether the probabilistic approach was used in this study.

 Other aspects of the protocol could benefit from clarification. For instance, it is not clear how the two climate datasets (ERA5-Land and BR-DWGD) will be integrated or reconciled; more detail would be helpful. The representativeness of SISVAN data, which appears to be limited to children engaging with the health system, raises concerns about selection bias.

 Finally, a dedicated limitations section would improve the transparency of the protocol, especially around spatial misclassification, data representativeness, and the ecological nature of some exposure assignments. Similarly, the growth and nutrition analyses should reflect the biological distinctions between wasting and stunting and their likely differing relationships with heat exposure across child age groups.

 In summary, this protocol represents an important and well-constructed study with the potential for significant impact. Incorporating spatially structured and spatially varying effects would substantially strengthen its methodological rigor and real-world relevance, and should be seriously considered as part of the core analytical strategy.

Is the study design appropriate for the research question?

Yes

Is the rationale for, and objectives of, the study clearly described?

Yes

Are sufficient details of the methods provided to allow replication by others?

Yes

Are the datasets clearly presented in a useable and accessible format?

Yes

Reviewer Expertise:

Spatial varying effect of exposure; Bayesian spatial modeling.

I confirm that I have read this submission and believe that I have an appropriate level of expertise to confirm that it is of an acceptable scientific standard, however I have significant reservations, as outlined above.

# Response

**Published:** 2025-07-31

**Authors:** Enny Paixao Cruz

**Affiliations:** Department of Infectious Disease Epidemiology & I, London School of Hygiene and Tropical Medicine, London, UK

This study protocol outlines a highly promising and timely investigation into the health impacts of heat exposure on birth outcomes, child nutrition, and feeding practices in Brazil. The research team leverages a remarkable array of linked administrative datasets, and the analytical plan demonstrates thoughtful consideration of time-varying exposures and lagged effects. The project is especially important in the context of low- and middle-income countries, where such large-scale evidence is scarce yet urgently needed.

 The proposed methodology is largely sound and appropriately ambitious, including use of distributed lag non-linear models (DLNMs), case-crossover designs, and time-to-event analyses. Thank you very much for your review

 However, one critical limitation is the absence of spatial statistical modeling. The current approach aggregates exposures at the municipality level, but this overlooks two important concerns: spatial correlation in both exposures and outcomes and how these correlations affect associations.

 To address this, the authors should consider spatially varying coefficient (SVC) models, ideally within the R-INLA framework, which is well-suited for large-scale hierarchical models with spatial components. Incorporating spatially structured random effects (e.g., using BYM2) or allowing exposure effects to vary geographically would offer two major advantages: First, it would account for spatial dependence and unmeasured area-level confounding; second, it would allow the team to detect regional variation in the heat-health relationship—variation that is likely to be highly relevant for targeted adaptation policy. Given the availability of rich spatial data through CIDACS, this is a missed opportunity and should be considered as and addition to the analysis strategy. Thank you for highlighting this issue. We plan to explore spatial correlation within a Bayesian framework in future analyses, particularly when evaluating small-areas and or taking advantage of the strengths of such model, as noted by the reviewer – e.g., for detecting regional variations. We also intend to evaluate the recently formalized Spatial-Bayesian DLNM approach (Quijal-Zamorano et al., 2024). However, due to the current lack of an exposure model with higher resolution and lack of better geocoding (beyond the municipality level), we have left it for a secondary analysis. For now, we have taken a more conservative approach by having strata by municipality. We agree this represents a missed opportunity and have acknowledged it as a limitation in the manuscript: “Furthermore, there are also limitations related to the exposure data. Temperature was assessed at the municipality level, which may lead to measurement error due to the inability to capture within-municipality variation, such as urban heat island effects. However, prior studies on mortality have shown that using city-level average temperatures produces results similar to those obtained with more spatially resolved models, suggesting that municipality-level exposure may still provide reasonable estimates. In future work, finer-scale exposure data – such as at the census tract level – may become available through the CIDACS Climate and Health Platform, offering opportunities for more spatially refined analyses, exploring spatial correlation within a Bayesian framework, such as the recently formalised spatial Bayesian DLNM approach.” ll. 466-476  

**#9** The authors state: "Two approaches have been employed to perform record linkage between the data sources: (a) deterministic linkage and (b) probabilistic linkage based on a similarity index." Only the deterministic linkage method appears to be described in the protocol; it would be helpful to clarify whether the probabilistic approach was used in this study. Thank you for this. We have now described both approaches in the manuscript. “The deterministic approach uses a unique identifier common in the two linked datasets. For example, climatic data from the CIDACS Climate and Health Platform was incorporated into the cohort database through deterministic linkage, using the municipality code of the mother's residence as a common identifier between datasets. To link the CIDACS Birth Cohort baseline with the SISVAN dataset, deterministic linkage was performed using a unique identifier available in both datasets – the Social Information Number (NIS, Número de Identificação Social). For records with missing NIS data, a non-deterministic linkage approach, similar to the method used to create the CIDACS Birth Cohort, was applied. The non-deterministic linkage process used to create the CIDACS Birth Cohort is described in detail by Almeida et al. (28). Matching criteria included variables such as maternal name, date of birth or age, and place of residence. This linkage was conducted using CIDACS-Record Linkage, an innovative tool developed at the Center for Data and Knowledge Integration for Health (CIDACS-RL) (29). CIDACS-RL utilizes a combination of indexing and search algorithms to identify records in the CIDACS Birth Cohort that closely match each record in the other datasets. The tool conducts pairwise comparisons of candidate linking records, ranks them based on similarity scores, and retains the highest-scoring pair as the potential link. The accuracy of each linkage will be assessed and described through manual verification of a random sample of records, evaluating sensitivity and specificity indexes via receiver operating characteristic curves.” ll. 257-275

**#10 **Other aspects of the protocol could benefit from clarification. For instance, it is not clear how the two climate datasets (ERA5-Land and BR-DWGD) will be integrated or reconciled; more detail would be helpful. Please see our response to comment #3

**#11 **The representativeness of SISVAN data, which appears to be limited to children engaging with the health system, raises concerns about selection bias. Please see our response to comment #2

**#12 **Finally, a dedicated limitations section would improve the transparency of the protocol, especially around spatial misclassification, data representativeness, and the ecological nature of some exposure assignments. Please see our response to comment #5

**#13 **Similarly, the growth and nutrition analyses should reflect the biological distinctions between wasting and stunting and their likely differing relationships with heat exposure across child age groups. Please see our response to comment #6 In summary, this protocol represents an important and well-constructed study with the potential for significant impact. Incorporating spatially structured and spatially varying effects would substantially strengthen its methodological rigor and real-world relevance and should be seriously considered as part of the core analytical strategy. Thank you.

# Reviewer report: Reviewer response for version 1

**DOI:** 10.21956/wellcomeopenres.26377.r121740

**Published:** 2025-05-19

**Authors:** Ana Bonell

**Affiliations:** 1 London School of Hygiene and Tropical Medicine Faculty of Epidemiology and Population Health, London, UK

This study protocol details the use of extensive  datasets from Brazil to explore the association between high temperature exposure and the following: birth outcomes (LBW, preterm birth, SGA, LGA); child growth (HAZ, WHZ,WAZ); and child feeding practices. The datasets are explained with the planned linkages between them. This is a well-written and well explained study protocol. The outcome of these studies will be important in understanding the risk of pregnant women and infants to extreme heat. There are a few minor points to consider:

 1. Exposure determination is based on area-weighted average values for the municipality. It appears that individual residence data is available in CIDACS (as indicated by the linkage of datasets process). Could individuals rather than municipality be linked to climate exposure? If this is not possible then are there plans to undertake a sensitivity analysis to understand if the described method would misusing exposure, for example due to the heat island effect?

 2. It is unclear if the SISVAN survey could introduce bias. Is the data only collected when individuals attend a health centre? If this is the case then it would be important to understand the implications of this?

 3. It is unclear how the two climate datasets will be used - both seem to have good coverage of the country.

 4. In the conceptual framework there is the following statement: " *During the second and third trimesters,* *exposure to higher ambient temperatures may increase the maternal *core body temperature, leading to a reduction in maternal metabolic rate, contributing to an increase in birth weight." Please expand the physiological reasoning behind this statement, since it is not clear how a reduction in maternal metabolic rate would result in an increased birth weight.

 5. Please add a limitations section.

 6. For the nutritional status/growth analysis - is this planned over a specific age range? Please consider the high likelihood that heat effects growth differently at different ages (https://www.thelancet.com/journals/lanplh/article/PIIS2542-5196(24)00208-0/fulltext). In addition, the analysis plan does not expand on the potentially different biological and environmental pathways found in the association between extreme heat and wasting versus stunting. Please explain how this will be taken into account.

 7. For the food intake/breast-feeding analysis - is the same analytical approach being taken? Please add a little detail on this study.

Is the study design appropriate for the research question?

Yes

Is the rationale for, and objectives of, the study clearly described?

Yes

Are sufficient details of the methods provided to allow replication by others?

Partly

Are the datasets clearly presented in a useable and accessible format?

Not applicable

Reviewer Expertise:

Maternal health and climate change.

I confirm that I have read this submission and believe that I have an appropriate level of expertise to confirm that it is of an acceptable scientific standard, however I have significant reservations, as outlined above.

# Response

**Published:** 2025-07-31

**Authors:** Enny Paixao Cruz

**Affiliations:** Department of Infectious Disease Epidemiology & I, London School of Hygiene and Tropical Medicine, London, UK

This study protocol details the use of extensive datasets from Brazil to explore the association between high temperature exposure and the following: birth outcomes (LBW, preterm birth, SGA, LGA); child growth (HAZ, WHZ, WAZ); and child feeding practices. The datasets are explained with the planned linkages between them. This is a well-written and well explained study protocol. The outcome of these studies will be important in understanding the risk of pregnant women and infants to extreme heat. There are a few minor points to consider: Thank you very much for your review. **#1 **Exposure determination is based on area-weighted average values for the municipality. It appears that individual residence data is available in CIDACS (as indicated by the linkage of datasets process). Could individuals rather than municipality be linked to climate exposure? If this is not possible then are there plans to undertake a sensitivity analysis to understand if the described method would misusing exposure, for example due to the heat island effect? We thank the reviewer for this thoughtful comment. As noted, our exposure assessment is currently based on area-weighted averages at the municipality level, which is consistent with the approach used in prior studies in Brazil (e.g., Chen et al., 2025; Requia et al., 2023). Although individual residence data are available in the CIDACS Birth Cohort, they have not yet been fully processed (i.e., geocoded) for analysis. Similarly, granular climate exposure data (e.g., at the census tract level) are also not yet available. These data are expected to be released through the CIDACS-Clima Climate and Health Platform in the future. Once accessible, we plan to apply finer-scale linkage approaches to better capture intra-municipality variation, including urban heat island effects. In the Brazilian context, municipalities are highly heterogeneous – often encompassing both urban and rural areas and varying considerably in size, population density, and infrastructure. We plan to conduct sensitivity analyses stratified by municipality size and level of urbanization. However, we will interpret any observed differences with caution, as they may reflect genuine effect heterogeneity rather than exposure misclassification. As suggested, we have added a limitation section which includes a discussion of the risk of exposure misclassification due to the use of municipality-level averages. “Furthermore, there are also limitations related to the exposure data. Temperature was assessed at the municipality level, which may lead to measurement error due to the inability to capture within-municipality variation, such as urban heat island effects. However, prior studies on mortality have shown that using city-level average temperatures produces results similar to those obtained with more spatially resolved models, suggesting that municipality-level exposure may still provide reasonable estimates. In future work, finer-scale exposure data – such as at the census tract level – may become available through the CIDACS Climate and Health Platform, offering opportunities for more spatially refined analyses as well as analyses exploring spatial correlation within a Bayesian framework, such as the recently formalised spatial Bayesian DLNM approach.” **#2** It is unclear if the SISVAN survey could introduce bias. Is the data only collected when individuals attend a health centre? If this is the case then it would be important to understand the implications of this? This is an important point, and we appreciate the opportunity to clarify how SISVAN data are collected and the potential limitations involved. SISVAN is not a survey, but rather a national surveillance system that routinely monitors food consumption and the nutritional status of individuals receiving care through Brazil’s Unified Health System (SUS). Data are collected during routine visits to primary healthcare facilities. As the data reflect individuals who access health services, there is potential for selection bias related to healthcare-seeking behaviour. In particular, individuals who do not attend primary care – whether due to better health, access barriers, or other reasons – are not captured in SISVAN. Furthermore, individuals with malnutrition or those at risk of adverse nutritional outcomes are more likely to have multiple evaluations recorded in this dataset. In addition, approximately 68% of children in SISVAN are Bolsa Família beneficiaries. This is because participation in nutritional monitoring is a program conditionality for families with children aged 0 to 7 years. As a result, SISVAN data are skewed toward more socioeconomically vulnerable populations, which may limit generalizability to the broader child population. While SISVAN provides valuable data for understanding nutrition and growth among socially vulnerable children – who are often most at risk of climate-related health effects – any interpretation or extrapolation of results beyond this group should be made with caution. As with other secondary data sources not originally designed for research purposes, potential issues such as underreporting, missingness, and misclassification also warrant consideration. In our newly added limitation sections, we have acknowledged and further elaborated on these issues. We have also provided more information on SISVAN data on the methods section. “SISVAN is a national surveillance system designed to collect data on individual anthropometric indicators and food intake, based on information recorded by Primary Health Care professionals during routine services in the public healthcare system (SUS, Sistema Único de Saúde). These professionals are responsible for delivering healthcare and conducting nutritional surveillance across all stages of life, including monitoring the nutritional status of children enrolled in the Bolsa Família conditional cash transfer program (25) (26). Between 2008 and 2017, SISVAN coverage among the SUS-using population increased from 17.7% to 45.4%. Coverage of the total resident population rose from 14.3% to 34.6% over the same period (Silva, Silva et al. 2023). The main objective of SISVAN is to support the evaluation and development of public health nutrition policies. The dataset available at CIDACS includes 307,245,508 records from 59,724,164 individuals, covering the period from 2008 to 2021.” ll. 212-224 “First, some outcomes are only recorded when individuals seek healthcare. For example, SISVAN data on food consumption and nutritional status are collected during visits to primary care facilities, introducing potential selection bias related to healthcare-seeking behaviour.  Moreover, individuals with malnutrition or those at risk of adverse nutritional outcomes are more likely to have multiple evaluations recorded in this dataset and approximately 68% of children in SISVAN are Bolsa Família beneficiaries, due to a program conditionality requiring nutritional monitoring for children aged 0-7. This results in an overrepresentation of socioeconomically vulnerable groups, further limiting the generalisability of findings derived from SISVAN. More broadly, generalisability is also constrained by the composition of the CIDACS Birth Cohort, which includes only individuals registered in CadÚnico, representing the poorest half of the Brazilian population. Second, the use of administrative data restricts the availability of key covariates, such as maternal comorbidities, parental smoking, or household characteristics like access to air conditioning. Lastly, administrative data are subject to missingness, underreporting, and misclassification. For example, preterm births are estimated to be underreported by around 15% in SINASC, and gestational age may be misclassified due to reliance on the date of last menstrual period.” **#3 **It is unclear how the two climate datasets will be used - both seem to have good coverage of the country. Thank you for this observation. While both datasets provide strong national coverage, they serve complementary purposes in our study. The Brazilian Daily Weather Gridded Data (BR-DWGD), based on ground-station observations, is widely used in Brazil (e.g., Dias & Sentelhas, 2021; Silveira Marinho et al., 2020; Tomasella et al., 2022). Using a national data set which is recognised and trusted within Brazil will support engagement and with local policy and public health audiences. ERA5-Land, by contrast, is an internationally recognised reanalysis dataset that offers a broader range of climate variables and hourly temperature estimates. This allows for direct calculation of mean temperature, rather than approximating it from daily minimum and maximum values as with BR-DWGD. Including ERA5-Land enhances comparability with international climate and health studies. As we develop more specific analysis plans for each individual study, the choice of primary dataset will depend on the study’s objectives, data requirements, and intended audience. In addition, this decision will be informed by validation and comparison work currently being carried out by colleagues to assess the performance of each dataset. While these studies are not part of the present protocol, they will provide useful evidence to support dataset selection in future analyses. Both datasets may also be used in sensitivity analyses where appropriate. We have adjusted the manuscript to clarify this approach. “We will use two climatic datasets due to their complementary strengths. The choice of primary dataset for each analysis will depend on the specific study objectives, data needs, and target audience. The BR-DWGD is widely recognised within Brazil and supports engagement with national stakeholders, while ERA5-Land facilitates comparability with international studies. Ongoing validation work will also inform this decision, and both datasets may be used in sensitivity analyses where appropriate.” **#4 **In the conceptual framework there is the following statement: " *During the second and third trimesters, exposure to higher ambient temperatures may increase the maternal *core body temperature, leading to a reduction in maternal metabolic rate, contributing to an increase in birth weight." Please expand the physiological reasoning behind this statement, since it is not clear how a reduction in maternal metabolic rate would result in an increased birth weight. Since fetal weight gain typically reaches its peak around the 35 ^th^ week of gestation, exposure to elevated temperatures during this stage may intensify the typical growth trajectory, resulting in higher birth weight. This may reflect an effect of outdoor temperature on maternal vascular function, and hence, nutritional supply to the fetus (Lawlor et al., 2005) or may be linked to improved placental performance or metabolic adaptations triggered by heat stress during this key period of rapid fetal development (Chersich et al., 2020). Moreover, during periods of intense heat, the common recommendation for pregnant women to increase fluid intake to prevent dehydration may inadvertently encourage the consumption of both sugary and non-sugary beverages, raising concerns about potential impacts on both maternal and fetal health, such as an increased risk of gestational diabetes, excessive maternal weight gain, fetal macrosomia, and large for gestational age (LGA) infants (Wang et al., 2024). Additionally, particularly in the final weeks of pregnancy, pregnant individuals tend to reduce their levels of physical activity (Mitro et al., 2022; Ramón-Arbués et al., 2023), a reduction that may be further intensified on hotter days. We have now included this explanation in our manuscript: “The influence of elevated temperatures on placental capacity and blood flow remains an area of ongoing investigation, with various proposed mechanisms suggesting divergent – sometimes opposing – impacts on adverse birth outcomes such as SGA or LGA. Some studies report reduced placental blood flow and impaired placental function, potentially contributing to the risk of SGA births. In contrast, other studies suggest that exposure to high temperatures in late pregnancy may influence maternal vascular function and nutrient delivery to the fetus (Lawlor et al., 2005), or enhance placental performance and trigger beneficial metabolic adaptations, thereby ultimately supporting fetal growth and resulting in higher birth weight (Chersich et al., 2020). Additionally, during periods of extreme heat, pregnant individuals are often advised to increase fluid intake to prevent dehydration. However, this may inadvertently lead to greater consumption of both sugary and non-sugary beverages, raising concerns about potential adverse effects on maternal and fetal health, such as increased risk of gestational diabetes, excessive maternal weight gain, fetal macrosomia, and LGA births (Wang et al., 2024). Moreover, physical activity tends to decline in the later stages of pregnancy (Ramón-Arbués et al., 2023; Mitro et al., 2022), a reduction that may be further exacerbated by high temperatures (17, 18). Elevated ambient temperatures have also been associated with increased maternal leptin levels (19), which may contribute to higher birth weight (20). Given the mixed evidence and the ongoing nature of research in this area, our investigation will consider both SGA and LGA outcomes to capture the full spectrum of potential fetal growth responses to prenatal heat exposure.” ll. 138-160 **#5** Please add a limitations section. Thank you for the suggestion. We have added a limitations section addressing key issues such as selection bias, generalisability, and exposure misclassification: “This study relies on routinely collected administrative data, which enables national coverage and a large sample size, but also presents several limitations. First, some outcomes are only recorded when individuals seek healthcare. For example, SISVAN data on food consumption and nutritional status are collected during visits to primary care facilities, introducing potential selection bias related to healthcare-seeking behaviour.  Moreover, individuals with malnutrition or those at risk of adverse nutritional outcomes are more likely to have multiple evaluations recorded in this dataset and approximately 68% of children in SISVAN are Bolsa Família beneficiaries, due to a program conditionality requiring nutritional monitoring for children aged 0-7. This results in an overrepresentation of socioeconomically vulnerable groups, further limiting the generalisability of findings derived from SISVAN. More broadly, generalisability is also constrained by the composition of the CIDACS Birth Cohort, which includes only individuals registered in CadÚnico, representing the poorest half of the Brazilian population. Second, the use of administrative data restricts the availability of key covariates, such as maternal comorbidities, parental smoking, or household characteristics like access to air conditioning. Lastly, administrative data are subject to missingness, underreporting, and misclassification. For example, preterm births are estimated to be underreported by around 15% in SINASC, and gestational age may be misclassified due to reliance on the date of last menstrual period. Furthermore, there are also limitations related to the exposure data. Temperature was assessed at the municipality level, which may lead to measurement error due to the inability to capture within-municipality variation, such as urban heat island effects. However, prior studies on mortality have shown that using city-level average temperatures produces results similar to those obtained with more spatially resolved models, suggesting that municipality-level exposure may still provide reasonable estimates. In future work, finer-scale exposure data – such as at the census tract level – may become available through the CIDACS Climate and Health Platform, offering opportunities for more spatially refined analyses as well as analyses exploring spatial correlation within a Bayesian framework, such as the recently formalised spatial Bayesian DLNM approach.” ll.447-476 **#6 **For the nutritional status/growth analysis - is this planned over a specific age range? Please consider the high likelihood that heat effects growth differently at different ages (https://www.thelancet.com/journals/lanplh/article/PIIS2542-5196(24)00208-0/fulltext). In addition, the analysis plan does not expand on the potentially different biological and environmental pathways found in the association between extreme heat and wasting versus stunting. Please explain how this will be taken into account. We agree that the influence of heat on child growth likely vary by age, and we acknowledge the importance of considering age-specific physiological vulnerability (Principi et al., 2025). In our analysis, we will stratify children by age group to explore potential heterogeneity in the associations between heat exposure and nutritional outcomes, based on critical windows of developmental sensitivity (Edney et al., 2022). Within the manuscript, we discuss stratified analyses by infant age: “As the data allow, we will stratify by maternal characteristics (e.g., education level, race/ethnicity, gestational age at delivery), infant characteristics (e.g., sex, age and birth weight categories) and family characteristics (e.g., area of residence – rural/urban, beneficiary or not of social programs) and test for interaction effects with the exposure.” Given that heat exposure can have both acute and chronic effects on child nutrition, there is currently no consensus on the optimal lag period for assessing its impact on undernutrition. Some studies have proposed using a one-year lag, based on the rationale that a longer time window is better suited to capturing both immediate and cumulative effects of temperature, while also accounting for seasonal variations. Accordingly, we plan to use a 52-week lag period to assess the potential medium- to long-term association between ambient temperature exposure and children's nutritional status. **#7 **For the food intake/breast-feeding analysis - is the same analytical approach being taken? Please add a little detail on this study. For example, to estimate the effect of heat on breastfeeding duration, we will use linear regression models to estimate the effect size (β) and corresponding 95% confidence intervals (95% CI), using median temperature as the reference category.
